# The metal tolerance profile of *Thlaspi goesingense *is mimicked in *Arabidopsis thaliana *heterologously expressing serine acetyl-transferase

**DOI:** 10.1186/1471-2229-7-63

**Published:** 2007-11-28

**Authors:** John L Freeman, David E Salt

**Affiliations:** 1 Center for Plant Environmental Stress Physiology, Purdue University, Horticulture and Landscape Architecture Department. West Lafayette, Indiana 47907, USA; 2 Biology Department, Colorado State University, Program in Molecular Plant Biology, Fort Collins, Colorado 80523, USA

## Abstract

**Background:**

The Ni hyperaccumulator *Thlaspi goesingense *is tolerant to Ni ≅ Zn, ≅ Co and slightly resistant to > Cd. We previously observed that elevated glutathione, driven by constitutive activation of serine acetyltransferase (SAT), plays a role in the Ni tolerance of *T. goesingense*.

**Results:**

Here we show that the elevated shoot concentration of glutathione, previously shown to cause elevated Ni tolerance in *Arabidopsis thaliana *heterologously expressing *T. goesingense *mitochondrial serine acetyltransferase (SATm), also causes tolerance to Co and Zn while slightly enhancing resistance to Cd. The level of tolerance afforded to each metal is ranked Ni ≅ Co, > Zn > Cd. The Ni ≅ Co, > Zn tolerances are positively correlated with both the accumulation of glutathione (GSH) and the ability to resist the oxidative damage induced by these different metals. Based on the relative concentrations of each metal used a relatively low level of resistance to Cd was observed in both *T. goesingense *and *TgSATm *expressing lines and Cd resistance was least correlated to GSH accumulation.

**Conclusion:**

Such data supports the conclusion that elevated glutathione levels, driven by constitutively enhanced SAT activity in the hyperaccumulator *T. goesingense*, plays an important role in the Ni, Co and Zn tolerance of this and other Thlaspi species. The hyper-activation of S assimilation through SAT is an excellent strategy for engineering enhanced metal tolerance in transgenic plants potentially used for phytoremediation.

## Background

The natural phenomenon of heavy metal tolerance in specialized hyperaccumulator plants has sparked the interests of plant physiologists, plant ecologists and evolutionary biologists for over 50 years [1]. The first investigation into metal tolerance present in plant species was by evolutionary ecologists studying primary successionary species that can colonize areas of newly created metaliferous soils such as mine spoils [2]. In recent years, the understanding of the physiological basis of metal(loid) tolerance has increased due to the development of new analytical and molecular technologies [3,4]. Our research has focused on investigating the physiology of metal tolerance in metal hyperaccumulating plants, which are clearly hyper-tolerant to the metals they hyperaccumulate. However, certain *Thlaspi *hyperaccumulator species also display co-tolerance to metals not necessarily present in their native habitat [5]. Metal tolerance and metal hyperaccumulation are different but inter-related phenomena, and have been extensively reviewed [1,4,6,7]. In metal hyperaccumulators the genetic determinants of metal tolerance and accumulation segregate independently, confirming that different genes are responsible for these phenomena [8,9]. Additionally, transcriptional profiling has opened a window into the identity of the genetic changes driving metal hyperaccumulation, by uncovering sets of coordinated gene expression differences that likely control uptake, accumulation and tolerance in hyperaccumulatorsb [10-12]

Metal toxicity in plants is often caused by the production of reactive oxygen species (ROS) [13-17]. Reduced glutathione (GSH) a strong antioxidant can directly reduce some active oxygen species [18] and also provides the reducing equivalents for the ascorbate-GSH antioxidant cycle [16]. Previous biochemical comparisons between *T. goesingense *and the closely related non-accumulator *A. thaliana*, revealed that in *T. goesingense *constitutively elevated GSH levels, driven by higher activity of the enzyme serine acetyltransferase (SAT), are partly responsible for the elevated Ni tolerance observed in this species [19]. The heterologous expression of *T. goesingense SATm *in *A. thaliana *was observed to significantly increase GSH levels, mimicking those found in *T. goesingense *and consequently conferred increased resistance to Ni [19]. Elevated shoot levels of *O*-acetyl-L-serine, the product of SAT and a precursor for glutathione biosynthesis has recently been show to also correlate with Ni/Zn hyperaccumulation ability across numerous genera in the Brassicaceae family [20].

SAT and its product OAS have been shown to control the enzymes responsible for sulfate reduction and cysteine biosynthesis in plants [21]. The production of OAS and cysteine also limit the overall rate of glutathione biosynthesis and the maintenance of an elevated glutathione pool [22,23]. Sulfur assimilatory mechanisms and the production of GSH are known to be highly induced by both heavy metal treatments and oxidative stress [24,25]. While oxidative stress tolerances in plants have been increased using transgenic methods to produce elevated levels of OAS, Cys and GSH [26-28], relatively little was known about the effect similar increases might have on the metal tolerance of plants. Increases in these metabolites, through over expression of specific enzymes involved in their biosynthesis, were found to result in Ni tolerance in *A. thaliana *[19] and Cd tolerance in *B. juncea *[29].

Here we report our observations of the role of GSH in conferring multiple metal tolerances in *A. thaliana *heterologously expressing *TgSATm*. These results demonstrate intriguing similarities with the metal tolerance profile observed in the Ni hyperaccumulator *T. goesingense *with contains naturally enhanced GSH levels. Such data supports our conclusion that constitutively elevated GSH plays an important role in the multi-metal (Ni, Zn and Co), tolerance and Cd resistance observed in *T. goesingense *and *A. thaliana *heterologously expressing *TgSATm*.

## Results

### Metal tolerance profile of the Ni hyperaccumulator *T. goesingense*

**Figure 1:**
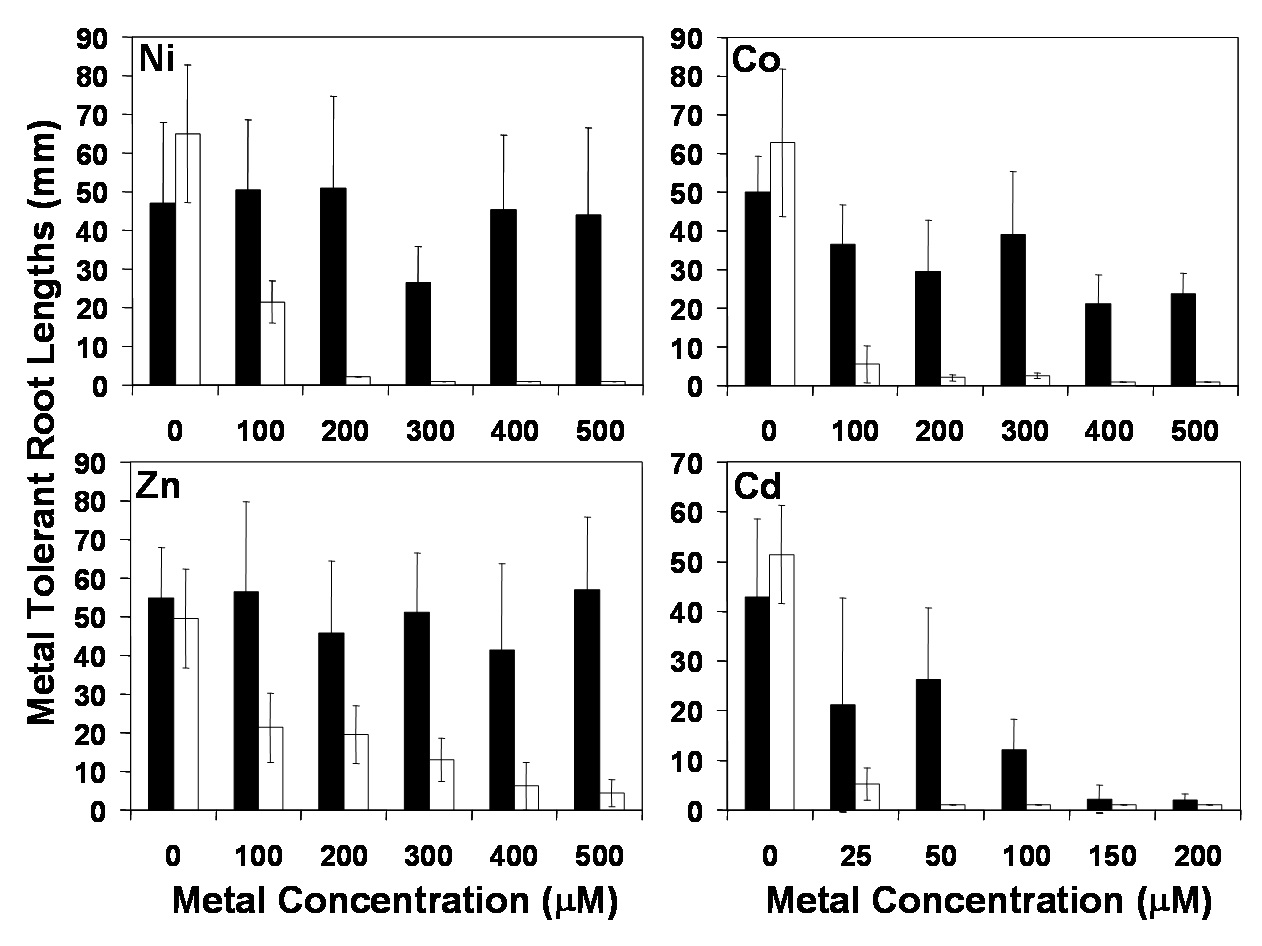
Metal tolerance present in *T. goesingense *(black) compared to *A. thaliana *(white) measured as root growth in (mm) 18 days after imbibition, on 1/2 strength MS medium containing 0 – 500 μM amounts of Ni (NO_3_)_2 _(**Ni**), Co (NO_3_)_2 _(**Co**), Zn (NO_3_)_2 _(**Zn**) and Cd (NO_3_)_2 _(**Cd**). (n = 6 *T. goesingense*. and *A. thaliana *plants for each bar ± SD).

**Figure 2:**
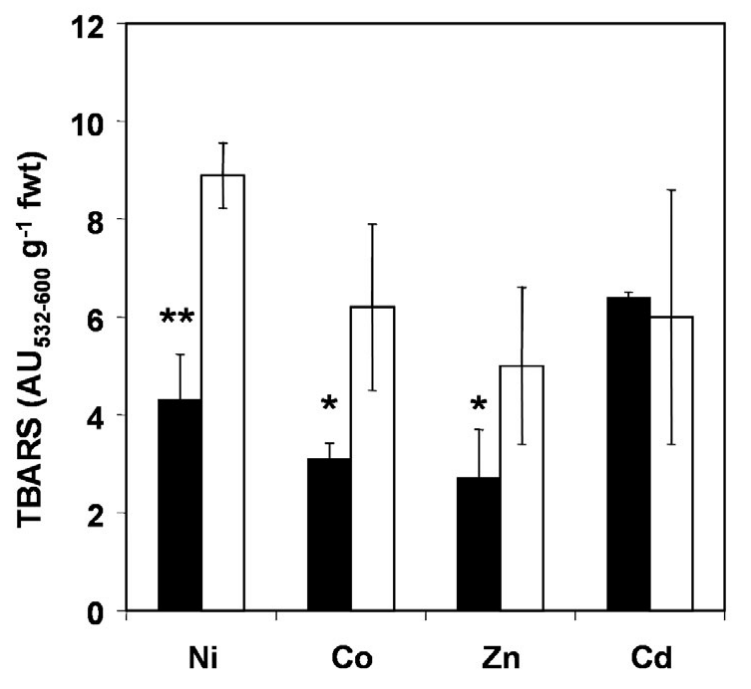
Shoot lipid peroxidation measured as TBARS in the hyperaccumulator *T. goesingense *(black) and the non accumulator *A. thaliana *(white) formed in response to each of the 4 different metal treatments after growth in normal media followed by transfer and growth on plates containing 1/2 strength MS medium with 125 μM Ni (NO_3_)_2_, 125 μM Co (NO_3_)_2_, 125 μM Zn (NO_3_)_2_, or 75 μM Cd (NO_3_)_2 _for a period of 8 days. Values represent difference in TBARS compared to growth on control media without added metals. Data represents averages (n = 4) ± SD. (*p < 0.05; ** p < 0.01, t-test)

Total GSH in mature *T. goesingense *and *A. thaliana *(Wassilewskija) was previously measured and found to be 412.5 ± 80 and 268.86 ± 48.1 nmol g^-1 ^fwt respectively [19]. The tolerance of *T. goesingense *to various metals including Ni, Co, Zn and Cd was investigated by measuring root growth compared to the non-tolerant relative *A. thaliana *Wassilewskija (WS) (Fig. 1) of plants grown on solidified MS medium containing Ni, Co, Zn or Cd. When comparing the concentration of a metal required for a 50% inhibition of root growth or (I_50_) *T. goesingense *was found to be the most tolerant to Ni ≅ Zn ≅ Co, with a low level of resistance to Cd (Table 1). The relative tolerance index (RTI) was calculated as the I_50 _*[T. goesingense]*/I_50 _*[A. thaliana]*. By using the RTI as a comparative measure of tolerance *T. goesingense *was observed to have enhanced relative tolerance to Ni ≅ Zn ≅ Co, with a low level of resistance to Cd when compared to *A. thaliana *(WS) (Table 1). To investigate how much of the metal tolerance observed in *T. goesingense *is due to increased resistance of oxidative damage we measured shoot lipid peroxidation in *T. goesingense *and *A. thaliana *after 8-days growth in the presence of Ni, Zn, Co or Cd (Fig. 2). We observed that relative to *A. thaliana *the hyperaccumulator *T. goesingense *contained significantly less peroxidized lipids after growth in the presence of Ni, Co and Zn, however no difference was observed after growth in the presence of Cd.

**Table 1:** Metal concentration (μM) which caused 50% inhibition of root growth (I^50^) for the different metals used on *T. goesingense *and *A. thaliana *followed by the relative comparative tolerance index of *T. goesingense *for different metals.

	Nickel (μM)	Cobalt	Zinc	Cadmium
*Thlaspi goesingense*	> 500	460	> 500	38
*Arabidopsis thaliana*	80	56	98	13
Comparative Tolerance Index (I^50 ^Thlaspi/I^50 ^Arabidopsis)	Ni > 6.28	Co 8.14	Zn > 5.09	Cd 2.84

### Metal tolerance profile of *A. thaliana *heterologously expressing *T. goesingense *serine acetyl-transferase

**Figure 3:**
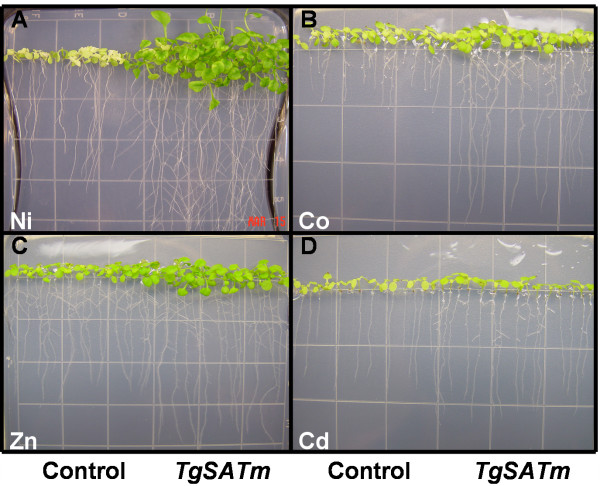
Vertical plates showing effect on metal tolerance of SAT ectopic expression in *A. thaliana *shoot tissue from homozygous T4 lines transformed with vector containing *TgSAT-m *(S 4–9) or empty vector (E 1–5) control. Plants were grown together on 1/2 strength MS upright agar plates containing 100 μM Ni (NO_3_)_2 _for 30 days from imbibition (**A**), plates containing 100 μM Co (NO_3_)_2_for 18 days from imbibition (**B**), 120 uM Zn (NO_3_)_2_, 18 days from imbibition (**C**), and 25 μM Cd (NO_3_)_2 _18 days from imbibition (**D**).

**Figure 4:**
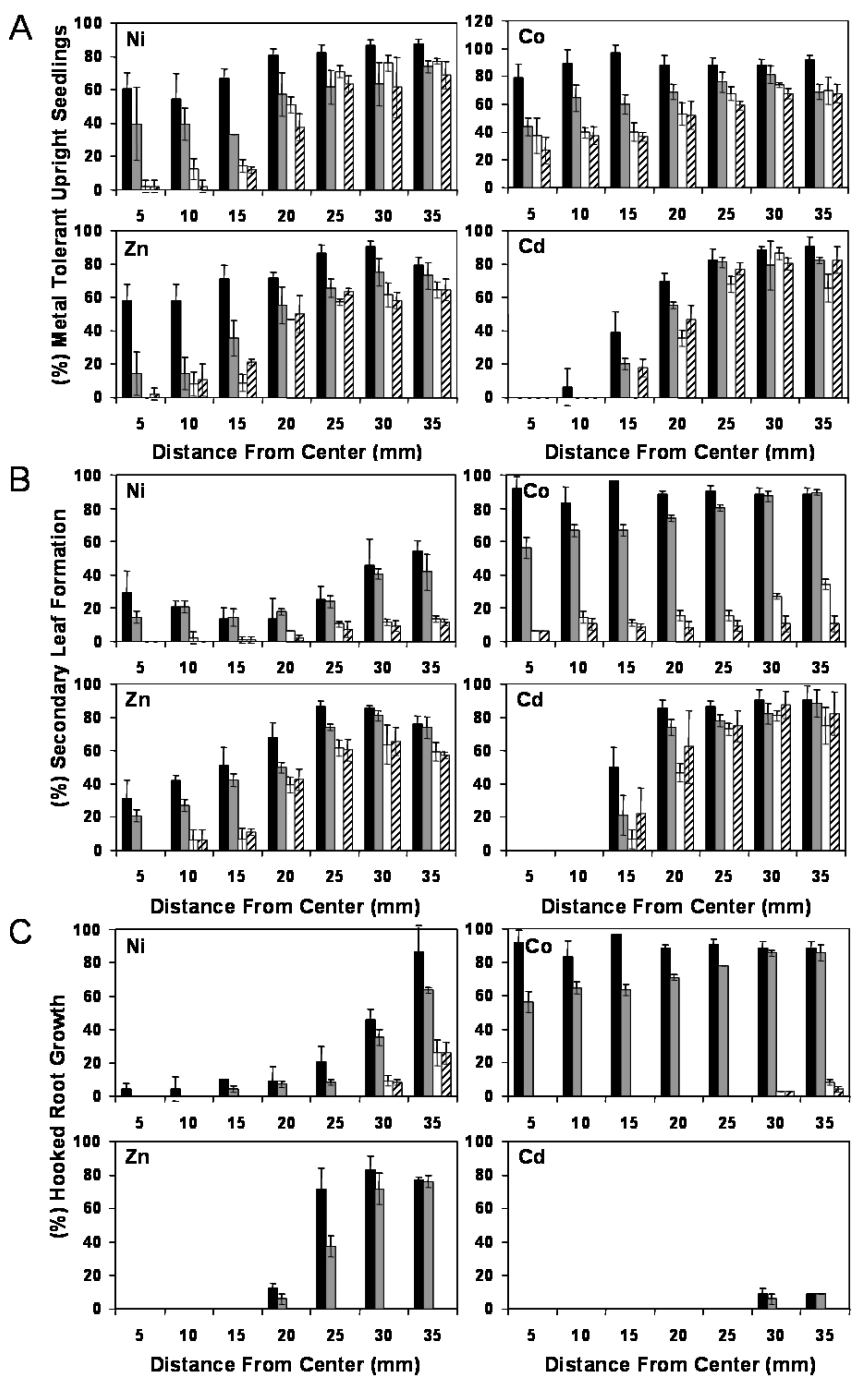
Metal tolerance caused by heterologous expression of Tg*SATm *in *A. thaliana*. Independent homozygous T4 lines transformed with *TgSAT-m *[S 4–9 (black), S 3-1 (grey) and S 5-4 (white)], representing high, intermediate and low expressers or empty vector E 1–5 (striped). Seedlings in each ring around a central filter paper disc soaked with 100 μL of 100 mM Ni (NO_3_)_2_, 100 mM Co (NO_3_)_2_, 400 mM Zn (NO_3_)_2 _and 50 mM Cd (NO_3_)_2_, were scored for upright cotyledons (**A**), development of secondary leaves (**B**) or the development of a root hook (**C**). Seedlings were germinated and grown for 13 days on the assay plates before being scored. Data represent the mean of three replicate assay plates per line ± SD.

Previously, we reported that heterologous expression of *T. goesingense *SAT (*TgSAT*_*m*_), leading to elevated shoot GSH levels, resulted in enhanced Ni tolerance of *A. thaliana *[19]. The total GSH present in *TgSAT-m *heterologous expressing lines, pooled from plates as young plants, was (S 4–9, 496 ± 20), (S 3-1, 369 ± 5), (S 5-4, 188 ± 9) nmol g^-1 ^Fwt, and represent the high, intermediate and low SAT expressors while the two empty vector controls with the same phenotype were (E 1–5, 172 ± 9) and (E 4–5, 142 ± 5) nmol g^-1 ^Fwt [19]. In addition to the *TgSATm *Ni tolerance shown growing in the vertical plates (Fig. 3A, 30 d), we also discovered metal tolerances to Co (Fig. 3B, 18 d) and to Zn (Fig. 3C, 18 d) and resistance to Cd (Fig. 3D, 18 d). The Ni tolerance of *TgSATm *was previously quantified using a metal growth, zone of inhibition assay, on plates, [19] (Fig 4A Ni). Using the same disc assay here, we quantify that plants heterologously expressing *TgSAT*_*m *_are also tolerant to Co (Fig. 4B) and Zn (Fig. 4C), and show enhanced resistance to Cd (Fig. 4D). By using lines with measured levels of *TgSAT*_*m *_expression, enzyme activity and GSH accumulation [19], and multiple empty vector controls, quantitative data was obtained for the growth response to Ni, Co, Zn and Cd of high S 4–9(black), intermediate S 3-1(grey) and low S 5-4(white) or non-expressing lines, empty vector E 1–5(striped), of *A. thaliana *transformed with *TgSAT*_*m*_. Though such an assay produces only a relative measurement of tolerance, quantified as the distance from a metal-soaked disc, it allows highly reproducible results and measurements of both tolerance and accumulation over a continuous gradient of metal concentrations. In this method we use different parameters to assess plant growth than just root lengths, including development of normal upright shoot cotyledons, secondary leaf formation, and formation of a root hook as the root bends on contact with the base of the Petri dish. Data is expressed as a percentage of the total number of seedlings in each ring around the metal soaked disc. This assay takes full advantage of the metal concentration gradient established as the metal diffuses from the disc, allowing resolution of differences in metal tolerance between lines and various metals. Based on estimations of shoot and root growth on both vertical plates and disk assay plates (Fig. 3 &4), heterologous expression of *TgSAT*_*m *_was observed to provide significant increases in tolerance to Ni, Co and Zn along with a slight amount of Cd resistance.

Shoot material from the *TgSATm *lines and empty vector controls grown at 20 mm and 35 mm from the disc were harvested for ICPMS analysis to investigate the respective metal concentrations. Interestingly, although tolerance to Ni, Co and Zn was increased in lines heterologously expressing *TgSAT*_*m*_, these tolerant lines had little or no increase in shoot accumulation of Ni, Co or Cd. The only exception to this was for Zn, because both the highest (S 4–9), and intermediate (S 3-1) SAT expressing and GSH accumulating *TgSATm *lines harvested from the 20 mm ring had a 1.3–1.5 fold higher shoot Zn concentration (8,936 ± 760 & 10,050 ± 458 μg Zn g^-1 ^Dwt respectively) compared to the low (S 5-4) and non-expressing line (E 1–5) at (6,704 ± 656 & 6,644 ± 94 μg Zn g^-1 ^Dwt respectively). While the tolerant SAT lines did not accumulate more metal than the others except for Zn the overall levels were all quite high, for Ni (~1,200), Zn (~6,000–10,000), Co (~2,000) and Cd (~2,000) μg g^-1 ^Dwt.

**Figure 5:**
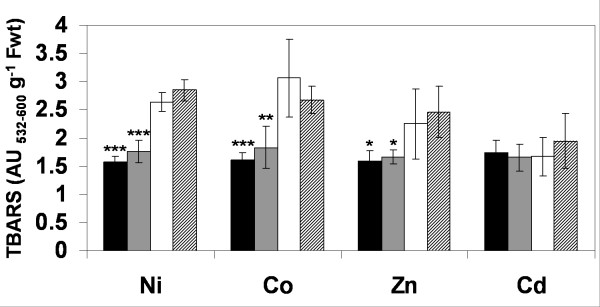
Effect of heterologous *TgSATm *expression on oxidative stress induced by different metals in *A. thaliana *shoot tissues from independent homozygous T4 lines transformed with vector containing *TgSAT-m*, S 4–9(black), S 3-1(grey) and S 5-4(white) or empty vector E 1–5(striped). Total shoot lipid peroxidation was measured as TBARS formed in response to each of the 4 different metal treatments after growth in normal media followed by transfer into 1/2 strength MS medium plates also containing 125 μM Ni (NO_3_)_2_, 125 μM Co (NO_3_)_2_, 125 μM Zn (NO_3_)_2_, and 75 μM Cd (NO_3_)_2 _for a period of 4 days. Values represent difference in TBARS compared to growth on control media without metals. Data represents averages (n = 6) ± SD (*p < 0.05; ** p < 0.01; *** p < 0.001, t-test).

To determine if enhanced resistance to oxidative stress plays a role in the metal tolerances conferred by heterologous expression of *TgSAT*_*m*_we measured lipid peroxidation in shoots of plants after growth in the presence of toxic levels of Ni, Co, Zn and Cd (Fig. 5). These results demonstrate that under the conditions used growth in the presence of Ni, Co and Zn produce greater lipid peroxidation than Cd. They also show that lines with relatively strong expression of *TgSATm *(black bars), have the lowest levels of lipid peroxidation in plants grown in the presence of either Ni, Co or Zn. However, heterologous expression of *TgSATm *conferred no decrease in lipid peroxidation in Cd exposed plants, most likely because Cd-induced lipid peroxidation was low even in control lines (Fig. 5).

**Figure 6:**
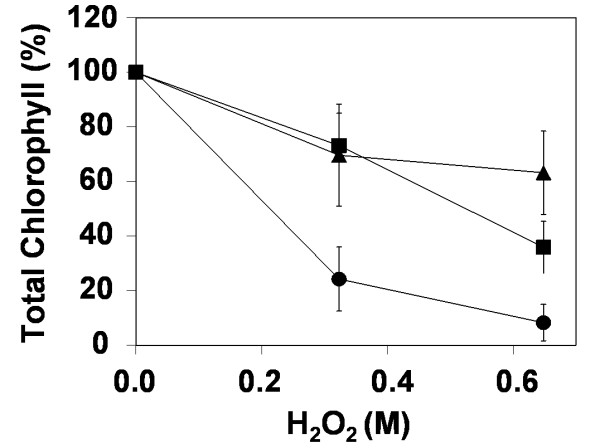
Oxidative stress resistance measured as total chlorophyll content in leaf discs of *T. goesingense *(squares), *A. thaliana *heterologously expressing *TgSATm *(triangles) and empty vector control *A. thaliana *(circles) after 24 hours flotation on hydrogen peroxide under high light. Data represents averages (n = 9 lf discs, 3 discs per plant, n = 3 plants) ± SD.

Heterologous expression of *TgSAT*_*m *_was also observed to increase resistance to hydrogen peroxide (Fig. 6). This assay measures the remaining chlorophyll content of leaf discs floated on various concentrations of hydrogen peroxide under high light. Both wild type and empty vector *A. thaliana *were used as controls and produced the same results, data for empty vector control is shown (Fig. 6). These results establish that heterologous expression of *TgSAT*_*m *_also enhances tolerance to general oxidative stress, beyond that caused specifically by growth in the presence of the metals tested. Interestingly, the hyperaccumulator *T. goesingense*, which is known to have constitutively elevated SAT activity and accumulate elevated GSH [19], also showed significant tolerance to hydrogen peroxide treatments (Fig. 6).

### Association of GSH with tolerance to Ni, Co, Zn and enhanced resistance to Cd

**Figure 7:**
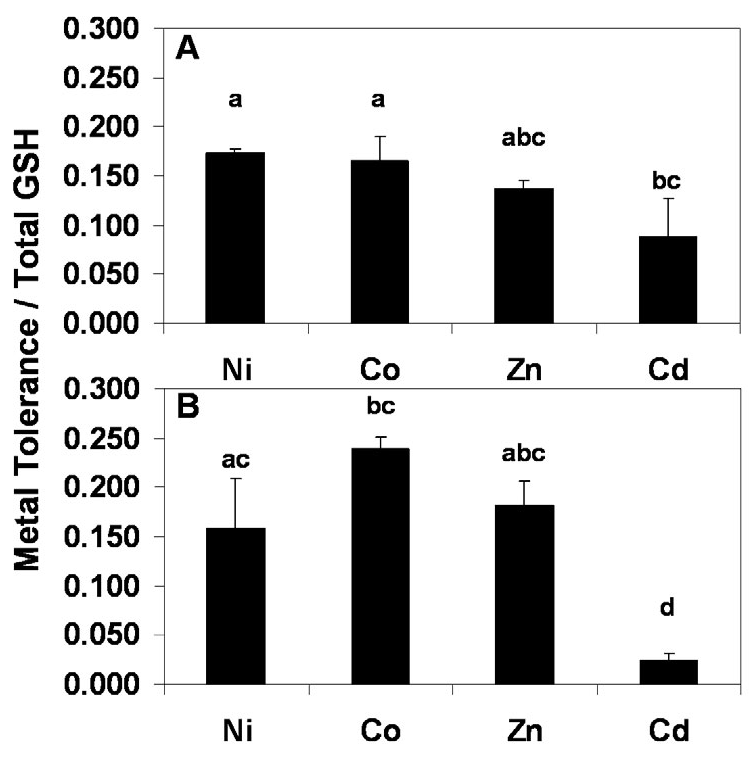
Bar graph showing the slopes of trend lines obtained through plotting metal tolerance to Ni, Co, Zn and Cd for each line against shoot GSH accumulation. Metal tolerance was scored as the percentage of seedlings with normal upright cotyledons (**A**) or the formation of a root hook (**B**). Data represent means of slopes (n = 3 for each metal ± SD). Lower case letters (a, b, c and d) represent significantly different means, using the Tukey-Kramer test comparing all pairs (P < 0.05). Fits of the metal tolerance verses GSH plots for Ni, Co, Zn and Cd in shoots, R^2 ^= 0.99, 0.96, 0.98, and 0.99, respectively, and for roots R^2 ^= 1.0, 1.0, 0.98 and 1.0.

Heterologous expression of *TgSAT*_*m *_was previously shown to increase both shoot GSH levels and Ni tolerance (Freeman et al., 2004). We observe here that in addition to increased Ni tolerance, elevated GSH also leads to increased tolerance to the growth inhibitory effects of Co, Zn and a low level of resistance to Cd. However, by comparing the magnitude of the tolerance to Ni, Co, Zn, and Cd resistance conferred by heterologous expression of *TgSAT*_*m *_with the concentration of shoot GSH, across lines with different levels of SAT expression, we established that the effectiveness of GSH in enhancing metal tolerance varies with the metal. The percent toxicity, scored for both shoot and root, was calculated for each line and metal at the seedling ring, which had the maximum difference in tolerance when the highest *SATm *expressing line was compared to the control line. These tolerance values were plotted against the GSH synthesis capacity of each line, and the relationship was found to be linear, with increasing GSH conferring increasing metal tolerance to Ni, Co, Zn, and resistance to Cd (DNS). From the slope of these plots the metal tolerance vs GSH concentration value is obtained for each metal in both shoot and root (Fig. 7). The equivalent increase in shoot GSH concentrations was most effective at increasing shoot tolerance to Ni and Co, followed closely by Zn then least effective was Cd resistance. GSH also caused elevated metal tolerances in roots to Co followed by Ni and Zn, while Cd resistance in roots was least correlated to total GSH production. However, metal levels in the roots were not measured and Cd may have been much higher here, as Cd is known to accumulate in roots.

## Discussion

The *T. goesingense *mitochondrial version of SAT was chosen for use in this study over that of the chloroplast and the cytosolic versions because it conveyed a high level of Ni tolerance to *A. thaliana *[19], and because of its high sequence similarity (95.0% identity) to the *A. thaliana *mitochondrial homolog At3g13110 [19]. Additionally, in *E. coli*, *TgSATm *conferred enhanced resistance to Ni [30], which was originally used to clone the three different SAT cDNAs from *T. goesingense *[19]. Furthermore, overexpression of the cytoplasmic, mitochondrial, or chloroplastic SAT from *T. goesingense *or the chloroplastic SAT from *A. thaliana *in *E. coli *all conferred approximately equal tolerance to Ni and Co [30]. The mitochondrial isoform of SAT (TgSAT-m) was also chosen for overexpression in this study because overexpression of the Cys-insensitive *A. thaliana *homolog AtSAT-m (SAT A) had been shown to achieve significant elevations in Cys and GSH concentrations, when overexpressed in tobacco and targeted to either the plastid or the cytosol [21]. In addition to making more OAS, Cys and GSH in the mitochondria, which can then be exported into the cytosol and other organelles, over-expression of TgSATm in the mitochondria could lower Ni induced ROS formation, so the electron transport chain can still provide cells with the energy needed to function.

The hyperaccumulator *T. goesingense *has significant tolerance to the growth inhibitory effects of elevated Ni, Zn and Co, with a slight resistance to Cd (Fig. 1 and Table 1), and in part this appears due to an enhanced ability to resist the oxidizing effects of Ni, Zn and Co (Fig 2). The elevated levels of GSH in *T. goesingense *are thought to contribute to the Ni tolerance of this plant, via enhancing resistance to the oxidizing effects of this metal [19]. We hypothesize that this elevated GSH also causes the elevated tolerance to Co and Zn, but only low level resistance to Cd. To address this question we utilized transgenic *A. thaliana *heterologously expressing *TgSAT*_*m *_that had previously been characterized to contain elevated shoot concentrations of GSH that confer enhanced resistance to Ni [19].

In addition to Ni our analysis of the metal tolerance of these same plants showed them to also have tolerance to growth in the presence of elevated Co and Zn while causing a low level of resistance to Cd, when compared to the control lines (Fig. 3 &4). Interestingly, the high and intermediate *TgSATm *expressing lines were found to accumulate ~1.4 fold more Zn when compared to the low and non-expressing lines. This suggests that increasing GSH and thus Zn tolerance might positively influence the Zn accumulation mechanisms in plants.

Furthermore, these transgenic plants also showed increased resistance to the lipid peroxidizing effects of Co and Zn (Fig. 5), suggesting that at least one of the roles of GSH in this enhanced Co and Zn resistance is as an antioxidant. These results mirror those previously observed for Ni [19]. However, the elevated GSH concentration in these lines had no effect on levels of lipid peroxidation in plants grown in the presence of Cd, suggesting that GSH is conferring enhanced tolerance to Cd via an unknown alternative pathway potentially through an increased ability to synthesize phytochelatins (PC). Cd resistance was quite low considering that both *T. goesingense *and the *TgSATm *could tolerate growth on plates containing at least 4 times higher concentrations of Ni, Co and Zn compared to Cd.

Our results establish that heterologous expression of *TgSAT*_*m *_also enhances tolerance to other oxidative stresses caused by hydrogen peroxide and high light treatment (Fig. 6). The hyperaccumulator *T. goesingense*, which is known to have constitutively elevated SAT activity and accumulate, elevated GSH [19], also shows high tolerance to hydrogen peroxide treatment (Fig. 6).

Analysis of the ability of GSH to confer metal tolerance in these transgenic lines also revealed that in shoots GSH is most effective at conferring tolerance to Ni = Co > Zn > Cd (Fig. 7A). This activity closely follows the levels of lipid peroxidation produced by growth on these three metals (Fig. 5). GSH production was least effective at conferring tolerance to Cd (Fig. 7A), which was exemplified by the no reduction in lipid peroxidation after Cd treatment (Fig. 5). In roots GSH conferred tolerance to Co > Ni = Zn > Cd (Fig. 7B).

In both *T. goesingense *and the *TgSATm *transgenics, SAT enhanced Ni, resistance appears not to involve coordination by PC. This is fully supported by a complete lack of thiol coordination to Ni in these plants [19,31] and also in *E. coli *heterologously expressing the same gene [30]. Additionally, Zn is also not coordinated by PC or thiols in the Zn tolerant hyperaccumulator *T. caerulescens *[32]. It has also been determined genetically in the *A. thaliana *PCS null mutant *cad1*, which has no measurable PC, that PC are not involved in either Ni or Zn resistance in *A. thaliana *but are directly needed for Cd resistance [33-36]. While PC are clearly not involved in resistance to Ni and Zn in *A. thaliana*, Co also failed to activate *A. thaliana *PC synthesis in assays of purified AtPCS1 activity [37]. Therefore Given the chemical similarity between Ni and Co we can speculate that the enhanced Co resistance observed in the transgenic plants is due to the direct antioxidant effect of increased GSH.

Our findings establish a strong connection between elevated shoot GSH and enhanced tolerance to the lipid peroxidizing effects of Ni = Co > Zn in both *T. goesingense *and transgenic *A. thaliana *containing elevated GSH concentrations. Such a link is supported by the observation that elevated GSH levels in both transgenic *A. thaliana *and *T. goesingense *correlate with enhanced resistance to the peroxidizing effects caused by hydrogen peroxide and high light treatments.

## Conclusion

We therefore conclude that the multiple metal tolerances to Ni, Co and Zn observed in *T. goesingense *are due to the constitutively elevated shoot GSH levels observed in this hyperaccumulator. These multiple metal tolerances are mimicked in Arabidopsis through heterologous expression of the *TgSATm *gene which results in elevated GSH accumulation in leaves and enhanced oxidative stress resistance.

## Methods

### Plant plate growth

Plants were germinated and grown on plates containing 1/2 strength MS + Gamborgs B5 medium in 0.8% plant tissue grade agar alone or with the different metals under 16 hr white light (120 μmol m^-2 ^s^-1 ^of photosynthetic photon flux (PPF)). ICP-MS analysis of the different metal levels was performed on all dried shoot tissue as previously described (Lahner et al., 2003).

### Metal tolerance assays

Metal resistance in *A. thaliana *was tested on agar plates (100 × 25 mm) containing 1/2 strength MS + Gamborgs B5 medium in 0.8% plant tissue grade agar. Seeds from *A. thaliana *heterologously expressing T. goesingense *SATm *and empty vector control lines were spotted in concentric rings radiating from the center of the plate where a filter paper disc was placed. Onto the disc 100 μL of 100 mM Ni(NO_3_)_2_, 100 mM Co (NO_3_)_2_, 400 mM Zn (NO_3_)_2 _or 50 mM Cd (NO_3_)_2 _was pipetted and the plates incubated at 24°C/20°C, 10 h/14 h light/dark, 120 μmol m^-2 ^s^-1 ^PPF. After 13 days growth from imbibition the percentage of upright seedlings with fully expanded cotyledons, secondary leaves and hooked roots were recorded for each ring along with the distance in mm from the metal soaked filter disc. Seedlings from both 20 and 35 mm rings were then harvested for ICP-MS analysis of metal levels. Metal analysis was performed on dried shoot tissue as previously described [38].

### Hydrogen peroxide treatments

Hydrogen peroxide treatments involved cutting 1 cm diameter leaf discs from the center of intact mature leaves. Discs were floated on either H_2_O_2 _or water for 24 h at 24°C in constant light (180 μmol m^-2 ^s^-1 ^PPF). Percent total chlorophyll was measured at 654 nm following extraction in 96% ethanol [39], and normalized to chlorophyll observed in the water treated tissue. The chlorophyll contents for the control (H_2_O) treatments were approximately the same per gram fresh weight for all plants used. Total Chlorophyll mg g^-1 ^fwt.; *T. goesingense *= 1.55 ± 0.32, *A. thaliana *= 1.57 ± 0.13, Transgenic *TgSATm *= 1.58 ± 0.13.

### Quantification of total lipid peroxidation

Peroxidized lipids were assayed as the presence of thiobarbituric acid reactive species (TBARS). Plants were grown for 17 days under 16 hr white light (120 μmol m^-2 ^s^-1 ^PPF) on vertical agar plates containing 1/2 strength MS + Gamborgs B5 medium in 0.8% plant tissue grade agar. After 4 or 8 days transfer to 1/2 strength MS + Gamborgs B5 medium in 0.8% plant tissue grade agar plates, containing either Ni, Co, Zn or Cd, shoot tissue was harvested and assayed for TBARS as described previously [40]. During the period of metal exposure all plants remained viable and continued growing

### Root length inhibition

*T. goesingense *and *A. thaliana *plants were germinated and grown on vertical plates containing 1/2 strength MS + Gamborgs B5 medium in 0.8% agar with various concentrations of Ni, Co, Zn and Cd for 18 days under 16 hr white light (120 μmol m^-2 ^s^-1 ^PPF). The (I^50^) values were calculated by taking the metal concentrations in plates which caused 50% inhibition of root growth for the different metals used on both *T. goesingense *and *A. thaliana*. The maximum normal root growth was measured on the control plates for this experiment in the absence of any metal.

## Authors' contributions

DES conceived the experiments with major contributions from JLF. JLF wrote the paper with major contributions from DES. JLF was primarily responsible for carrying out the experiments. All authors have read and approved the final manuscript.
